# Nodal metastasis in cervical cancer occurs in clearly delineated fields of immune suppression in the pelvic lymph catchment area

**DOI:** 10.18632/oncotarget.5398

**Published:** 2015-09-28

**Authors:** A. Marijne Heeren, Eline de Boer, Maaike C.G. Bleeker, René J.P. Musters, Marrije R. Buist, Gemma G. Kenter, Tanja D. de Gruijl, Ekaterina S. Jordanova

**Affiliations:** ^1^ Center Gynecological Oncology Amsterdam (CGOA), Department of Obstetrics and Gynecology, VU University Medical Center, 1081 HV Amsterdam, The Netherlands; ^2^ Department of Medical Oncology, VU University Medical Center-Cancer Center Amsterdam, 1081 HV Amsterdam, The Netherlands; ^3^ Department of Pathology, VU University Medical Center, 1081 HV Amsterdam, The Netherlands; ^4^ Laboratory for Physiology, Institute for Cardiovascular Research, VU University Medical Center, 1081 BT Amsterdam, The Netherlands; ^5^ Center Gynecological Oncology Amsterdam (CGOA), Department of Obstetrics and Gynecology, Academic Medical Center, 1105 AZ Amsterdam, The Netherlands; ^6^ Center Gynecological Oncology Amsterdam (CGOA), Department of Gynecology, Netherlands Cancer Institute - Antoni van Leeuwenhoek, 1006 BE Amsterdam, The Netherlands

**Keywords:** Immunology and Microbiology Section, Immune response, Immunity, cervical cancer, tumor-draining lymph nodes, metastatic niche, tregs, PD-L1-myeloid cells

## Abstract

In cervical cancer, high frequencies of regulatory T cells (Tregs) and immunosuppressive PD-L1^+^CD14^+^ antigen-presenting cells dominate the microenvironment of tumor-positive lymph nodes (LN+). It is unknown whether this is restricted to LN+ or precedes metastasis, emanating from the primary tumor and spreading through tumor-draining lymph nodes (TDLNs). To investigate immunosuppression in the lymphatic basin of cervical tumors, all dissected TDLNs of five cervical cancer patients (in total 9 LN+ and 74 tumor-negative lymph nodes (LN−)) were analyzed for FoxP3^+^ Tregs, CD8^+^ T cells, HLA-DR^+^- and PD-L1^+^ myeloid cells by immunohistochemistry.

Tregs and PD-L1^+^ cells were found to form an immunosuppressive cordon around metastatic tumor cells. Importantly, whereas high HLA-DR^+^- and PD-L1^+^ cell rates were strongly associated with LN+, elevated Treg levels and decreased CD8^+^ T cell/Treg ratios were found similar in LN+ and adjacent LN−, as compared to LN− at more distant anatomical localizations. These data suggest that delineated fields of Treg-associated immune suppression in anatomically co-localized TDLNs enable metastasis by creating metastatic niches. This may be of importance for decision-making regarding (surgical) intervention in cervical cancer. Future efforts should include the implementation of immunotherapeutic regimens to overcome this immune suppression, establish loco-regional control and halt systemic tumor spread.

## INTRODUCTION

Cervical cancer is caused by a persistent infection with high-risk human papillomavirus (HPV) types, and is therefore an immunogenic disease which requires a highly immunosuppressive microenvironment in order to progress and metastasize [1]. Various immune suppressive cells are recruited, expanded and activated at the site of the primary tumor [2, 3]. These cells are able to inhibit and suppress activation of the immune system, and promote an immune suppressive microenvironment which supports tumor growth, not only in the primary tumor but also in tumor-draining lymph nodes (TDLNs) [4–6]. Recently, we reported on the presence and abundance of suppressive PD-L1^+^CD14^+^ M2-macrophage-like cells, myeloid-derived suppressor cells (MDSCs), T cells expressing co-inhibitory molecules (PD-1 and CTLA-4), and regulatory T cells (Tregs) in tumor-positive lymph nodes (LN+) compared to tumor-negative lymph nodes (LN−) from cervical cancer patients [7]. Tregs present in cervical TDLNs were previously found to be HPV-specific and functionally suppressive [8]. In line with the excess of suppressive immune cell subsets, decreased levels of interferon-γ (IFNγ) and high levels of interleukin-6 (IL-6), IL-10, and vascular endothelial growth factor (VEGF) were detected in cervical metastatic lymph nodes [7, 9]. This immune suppression in metastatically involved lymph nodes will stand in the way of effective anti-tumor immunity and may have to be tackled before immunotherapy can be effective and halt metastatic spread. In particular PD-L1 expressed on tumor-associated M2-like macrophages may represent an attractive therapeutic target [7, 10]. While systemic immune checkpoint blockade in cervical cancer is currently being explored in several clinical trials [11], more localized targeting of the microenvironment of cervical tumors and their TDLN may be even more effective while minimizing side effects [12].

Information is lacking on the localization and distribution of immune cell subsets in pelvic lymph nodes with respect to lymphatic drainage patterns and tumor involvement. Only one study focused on the difference between proximal and distal lymph nodes in relation to the primary tumor and reported a significantly higher CD4^+^/CD8^+^ T cell ratio in the proximal lymph nodes [13]. FoxP3^+^ Tregs and potentially immunosuppressive macrophage-like cells (expressing HLA-DR and PD-L1) are able to cross-talk and thereby mutually amplify their suppressive activity on antitumor (CD8^+^) T cells [14, 15]. One of the crucial check-point molecules involved in this process is PD-L1, since it can bind to PD-1 on T cells thereby inhibiting their function [16]. Therefore, in the present study we analyzed the distribution and localization of FoxP3^+^ Tregs, CD8^+^ T cells, HLA-DR^+^- and PD-L1^+^ myeloid cells in all pelvic lymph nodes, including LN+ and LN−, removed during primary surgery of five patients with cervical cancer. We show the presence of an immune suppressive cordon of Tregs and PD-L1^+^ cells around metastatic tumors in the TDLN and provide evidence for anatomically delineated fields of immune suppression characterized by high numbers of Tregs, in the tumor lymph catchment area, co-localizing with LN+ and suggestive of metastatic niche formation.

Cervical cancer is typically treated with radical hysterectomy and pelvic lymphadenectomy or chemoradiation [17–19]. Our findings argue in favor of intratumoral immune potentiation (e.g. pre-surgical or combined with chemoradiation) to stem immune suppressive lymph drainage from the tumor, avoid intranodal metastatic niche formation, and enable anti-tumor T cell activation.

## RESULTS

We studied the intranodal distribution and localization of FoxP3^+^ Tregs, CD8^+^ T cells, HLA-DR^+^- and PD-L1^+^ cells in five patients with cervical cancer by a triple FoxP3/CD8/HLA-DR immunofluorescence staining and immunohistochemical staining for PD-L1. HLA-DR^+^- and PD-L1^+^ cells were morphologically identified as myeloid-like cells, based on their dendritic cell (DC)- or macrophage-like shape. In the cervical lymph nodes we were able to identify several immune cell subsets, including single FoxP3^+^-, single CD8^+^-, single HLA-DR^+^- and PD-L1^+^ cells. In addition, we observed double positive FoxP3^+^HLA-DR^+^-, CD8^+^HLA-DR^+^- and CD8^+^FoxP3^+^ cells. However, only a few double positive immune cells were present per image, therefore we did not include these in our analysis.

### Distribution and localization of FoxP3^+^, CD8^+^ and PD-L1^+^ cells in LN+

**Figure 1:**
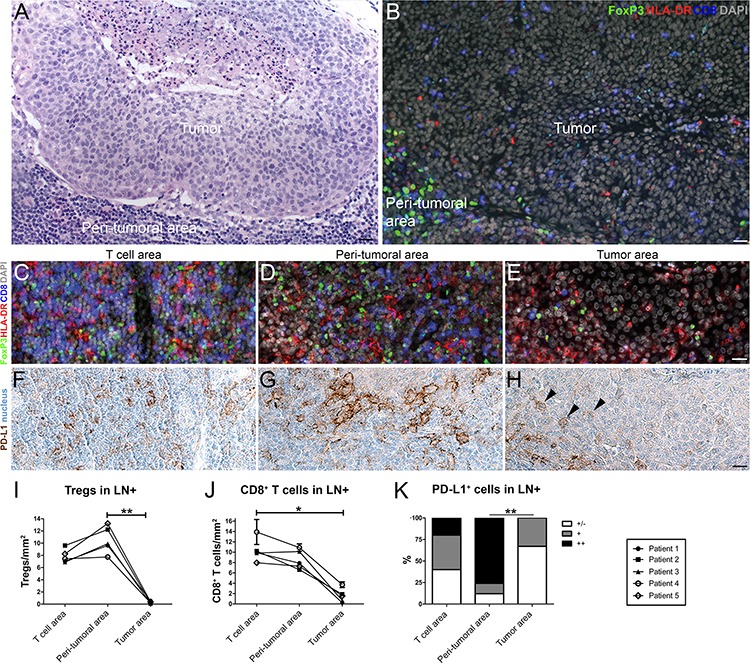
Tregs, CD8^+^ T cells and PD-L1^+^ myeloid cells in the paracortical T cell area, peri-tumoral and tumor area in metastatic lymph nodes **A.** H&E staining of a representative tumor-positive lymph node (LN+) showing the peri-tumoral and tumor area. Triple immunofluorescence staining of FoxP3 (green), HLA-DR (red) and CD8 (blue) of a **B.** LN+ (sequential section to the one shown in (A), magnification 100x, scale bar is 20 μm) showing the presence of Tregs, CD8^+^ T cells and HLA-DR^+^ cells in **C.** the paracortical T cell area, **D.** the peri-tumoral area, and **E.** the tumor area in a LN+. Nuclei are counterstained with DAPI (grey) (magnification 200x, scale bar 20 μm). In a sequential section, also PD-L1^+^cells (in brown) were present in **F.** T cell area, **G.** peri-tumoral area, and **H.** tumor area. Nuclei are counterstained with Hematoxylin (blue) (magnification 200x, scale bar is 20 μm), more **I.** Tregs, **J.** CD8^+^ T cells and **K.** PD-L1^+^ cells were observed in the peri-tumoral areas compared to the metastatic tumor fields. In (H), arrow heads indicate PD-L1^+^ infiltrating cells among the weakly positive tumor cells. In (I) and (J), the overall *P* value was calculated with One-way ANOVA Friedman test, and Dunn's post hoc test was used to compare individual groups. The Fisher Exact Test was used in (K) *P = 0.01 to 0.05 and **P = 0.001 to 0.01.

We studied the distribution and localization of Tregs, CD8^+^ T cells, single HLA-DR^+^- and PD-L1^+^ myeloid cells in all LN+. Nuclear DAPI stain was used to distinguish tumor tissue from normal tissue and in case of doubt a sequential Hematoxylin & Eosin (H&E)-stained slide was consulted (Figure 1A, 1B). A distinction between paracortical T cell area, peri-tumoral area and tumor area in LN+ was made (Figure 1A–1H), and we found a significant difference in the amount of Tregs per mm^2^ between the three areas (*P* = 0.008). Accumulation of Tregs was observed in the peri-tumoral areas, whereas limited numbers of Tregs were found in the metastatic tumor fields (*P* < 0.01) (Figure 1I). Furthermore, we found a significant difference in the amount of CD8^+^ T cells per mm^2^ between the three areas (*P* = 0.009), with higher numbers in paracortical T cell areas and only a few infiltrating the metastatic tumor area (*P* < 0.05) (Figure 1J). Moreover, we observed a significant difference in the distribution of PD-L1^+^ myeloid cells among the three areas (*P* = 0.038), with more PD-L1^+^ cells in peri-tumoral areas than in tumor areas (*P* = 0.017) (Figure 1K). Of note, metastatic tumor cells of 5/9 LN+ were weakly positive for PD-L1, however we were still able to identify PD-L1^+^ tumor infiltrating myeloid cells by the dense membranous PD-L1 expression compared to the relative dim expression on tumor cells (Figure 1H). Together these data point to a cordon of immune cells, heavily populated by Tregs and PD-L1^+^ myeloid cells around nodal metastases.

Anecdotally, we collected fresh samples of one LN+, including a sample of ‘white’ tissue, referred as ‘tumor-and peri-tumoral area’, and one sample of ‘dark’ tissue, referred as ‘T cell area’, macroscopically determined by an experienced pathologist. We studied CD4^+^ and CD8^+^ T cell ratios and Treg (identified by CD3^+^CD4^+^CD25^high^FoxP3^+^) frequencies in both samples by flow cytometry, and found in concordance with our immunohistochemistry data, a higher percentage of CD8^+^ T cells (46.2% vs. 25.1%) and a lower percentage of CD4^+^ T cells (48.1% vs. 72.3%) in the tumor area than in the T cell area. Additionally, we found more Tregs (12.5%) in the tumor area compared to the T cell area (2.8%) (Supplementary Figure 1).

### Patterns of immune suppression in the tumor lymph draining catchment area

**Figure 2:**
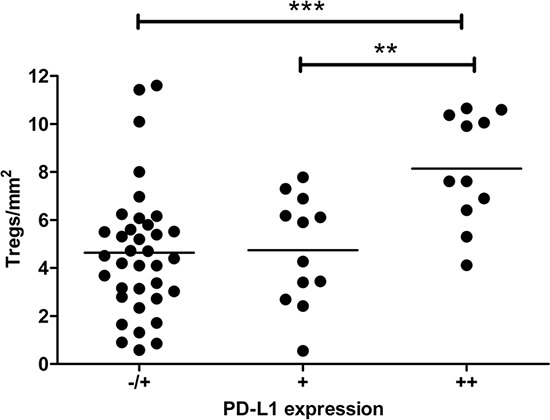
Association between high Treg- and high PD-L1^+^ myeloid cell rates in cervical lymph nodes Treg and PD-L1^+^ myeloid cell rates in non-tumor regions of all cervical lymph nodes were significantly correlated. The overall *P* value was calculated with One-way ANOVA Kruskal-Wallis test. For testing of selected groups, Dunn's post hoc test was used. **P = 0.001 to 0.01 and ***P < 0.001.

In a previous flowcytometry-based study, we found a significant correlation between Treg and PD-L1^+^ macrophage-like cell rates in single-cell suspensions from TDLN [7]. Here, we confirmed these findings: a significant association was found in the studied lymph nodes between high Treg frequencies and high PD-L1^+^ myeloid cell numbers in non-tumor regions. These regions were defined as paracortical areas in case of LN− and combined paracortical and peri-tumoral areas in case of LN+ (*P* = 0.003) (Figure 2).

**Figure 3:**
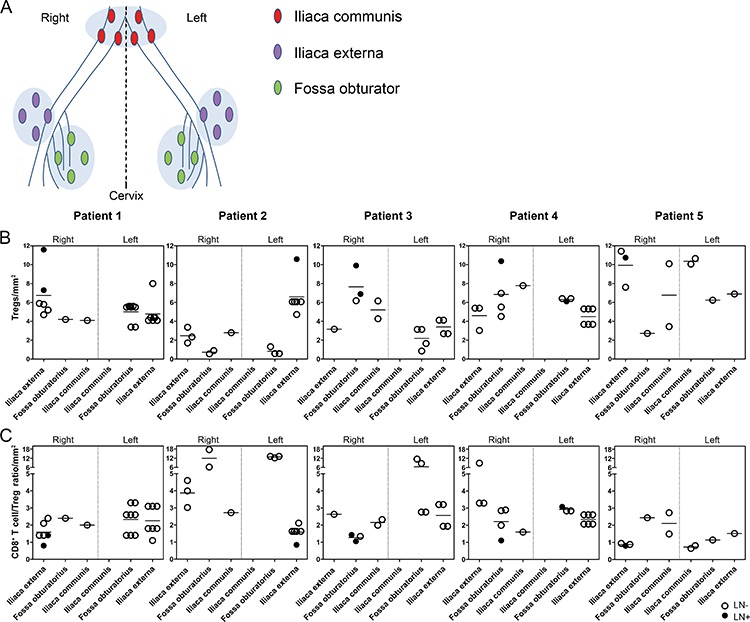
Treg levels and CD8 T cell/Treg ratios in the lymphatic basin of five patients with cervical cancer **A.** Reconstruction of the anatomical locations of pelvic lymph nodes from all patients, showing the following regions: iliaca externa (purple), fossa obturator (green) and iliaca communis (red) on both sides of the body (right and left). Graphs showing **B.** Treg levels per mm^2^ and **C.** CD8^+^ T cell/Treg ratios per mm^2^. Closed circles represent LN+, open circles represent LN−.

**Figure 4:**
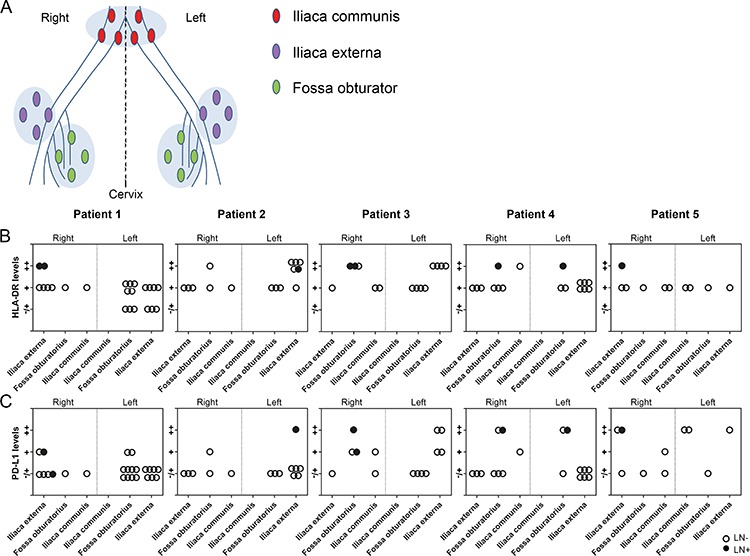
HLA-DR and PD-L1 levels in the lymphatic basins of five patients with cervical cancer **A.** Reconstruction of the anatomical locations of pelvic lymph nodes from five patients with cervical cancer, showing the following regions: iliaca externa (purple), fossa obturator (green) and iliaca communis (red) on both sides (right and left). Graphs showing **B.** HLA-DR levels and **C.** PD-L1 levels (in paracortical areas in tumor-negative lymph nodes (LN−) and in case of tumor-positive lymph nodes (LN+), paracortical and peritumoral areas) per lymphatic basin per patient. Levels for HLA-DR and PD-L1 are indicated by (−/+) minimal, (+) moderate, and (++) high numbers of positive cells. Closed circles represent LN+, open circles represent LN−.

Next, we investigated Treg, CD8^+^ T cell, HLA-DR^+^- and PD-L1^+^ myeloid cell numbers in paracortical areas, and in case of LN+, paracortical and peri-tumoral areas, in all lymph nodes that could be delineated according to succession in the lymphatic drainage of the primary tumor (from proximal to distal and therefore excluding parametrial lymph nodes) according to their anatomical position (iliaca externa left or right, fossa obturator left or right, and iliaca communis left or right, based on the pathology reports) (Figures 3A, 4A). This allowed for the identification of tumor-draining lymphatic patterns per patient, based on fields of immune suppression. We found evidence of a unique immune suppression-delineating draining pattern per patient, with varying levels of Tregs (Figure 3B), CD8^+^ T cell/Treg ratios (Figure 3C), and HLA-DR^+^- (Figure 4B) and PD-L1^+^ myeloid cells (Figure 4C) between LN. High Treg levels, low CD8^+^ T cell/Treg ratios, and high numbers of HLA-DR^+^ cells were consistently found in LN+ (Figures 3 and 4). High numbers of PD-L1^+^ cells were found in 5 out of 8 LN+, while in the remaining three LN+, minimal to moderate numbers of PD-L1^+^ cells were observed (Figure 4C), but this could be due to the fact that two of these LN+ had micro-metastases and one LN+ consisted almost entirely of metastatic tumor cells. Interestingly, 39.7% of the LN− had high Treg levels and 45.2% of the LN− had low CD8^+^ T cell/Treg ratios, whereas only 15.1% of the LN− had high numbers of HLA-DR^+^ cells, and 8.2% of the LN− had high numbers of PD-L1^+^ cells. Of note, as shown in Figures 3 and 4, these apparently affected LN− with high Treg rates most often co-localized with LN+ at the same side and in the same anatomical lymph node stations relative to the primary tumor, and thus seemed to delineate an immune suppressive lymph flow from the primary tumor, apparently facilitating loco-regional tumor spread.

**Figure 5:**
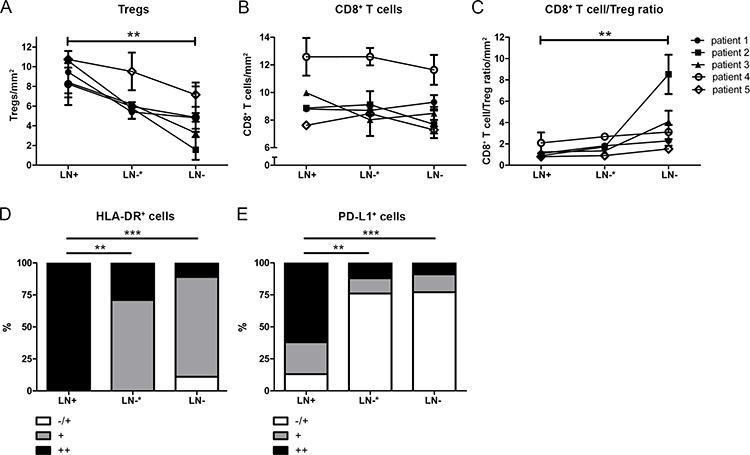
Frequencies of immunosuppressive cell subsets in tumor positive lymph nodes (LN+), proximal (to the LN+) uninvolved lymph nodes (LN−*) and more distal uninvolved lymph nodes (LN−) from patients with cervical cancer **A.** High levels of Tregs in tumor-positive lymph nodes (LN+) and tumor-negative lymph nodes (LN−), located in the same region as LN+ (LN−*), and significantly lower levels of Tregs in LN− compared to LN+. **B.** Constant levels of CD8^+^ T cells in LN+, LN−*, and LN−. **C.** Low CD8^+^ T cell/Treg ratios in LN+ and LN−*, and higher CD8^+^ T cell /Treg ratios were found in LN− compared to LN+. **D.** The distribution of HLA-DR^+^ cells in LN+ was significantly different compared to LN−* and LN−. **E.** The distribution of PD-L1^+^ cells in LN+ was significantly different compared to LN−* and LN−. Minimal- (−/+); moderate- (+); and high numbers (++) of positive cells. One-way ANOVA Friedman test *P*-value in (A), (B), and (C), Fisher's Exact test *P* value in (D) and (E) (**P = 0.001 to 0.01 and ***P < 0.001).

Subsequently, all lymph nodes from all patients were divided over three different groups: LN+, LN−* (present in the same anatomical location, i.e. same side and lymph node station, as the LN+) and all remaining LN− (i.e. not co-localized with LN+). Numbers of Tregs, CD8^+^ T cell/Treg ratios, HLA-DR^+^- and PD-L1^+^ myeloid cells were compared between these groups. No differences were observed for Treg numbers and CD8^+^ T cell/Treg ratios between LN+ and LN−*, whereas significant differences were observed between LN+ and LN−, confirming elevated Treg rates in LN− proximal to LN+. No significant differences were found for CD8^+^ T cell levels between the three different groups (Figure 5A, 5B, 5C). In contrast, for HLA-DR^+^- and PD-L1^+^ myeloid cells, significant differences were found both between LN+ and LN−* (*P* = 0.002 for HLA-DR and for PD-L1) and between LN+ and LN− (for both *P* < 0.001), but not between LN−* and LN− (Figure 5D, 5E), revealing a more strict tumor-associated recruitment of potentially suppressive PD-L1^+^ myeloid cells.

## DISCUSSION

Progression of cervical cancer manifests predominantly by local expansion and through lymphovascular space invasion, and is significantly associated with the risk of pelvic lymph node metastasis, which is an important indicator for poor prognosis [19, 20]. In this study, we investigated the localization and distribution of FoxP3^+^-, CD8^+^-, HLA-DR^+^- and PD-L1^+^ myeloid cells in all pelvic lymph nodes from five SCC cervical cancer patients with lymph node metastases. This is the first study to investigate the microenvironment of pelvic lymph nodes in different locations within the pelvic lymphatic basin, in order to study immune suppression in LN+ in relation to other proximal lymph nodes and determine suppressive lymphatic draining patterns per patient.

Our results point to a strong immunosuppressive microenvironment in LN+ from patients with cervical cancer with high Treg levels, low CD8^+^ T cell/Treg ratio, and high levels of PD-L1^+^- and HLA-DR^+^ myeloid cells, which is consistent with previous data based from flowcytometric analyses [7, 9] and immunohistochemical stainings [2]. In addition, we identified a peri-tumoral area with immunosuppressive FoxP3^+^ Tregs and PD-L1^+^ myeloid cells accumulation whereas only a minimal number of immune cells were observed infiltrating into the tumoral areas in lymph nodes from patients with cervical cancer [3, 21, 22]. Consistently, accumulation of immune cells surround metastases was also reported in another study of metastatic cervical lymph nodes and in melanoma LN+ [2, 23, 24]. This cordon around the metastases might be caused by the presence of immunosuppressive factors such as PD-L1, IDO, IL-6 and Prostaglandin-E2 (PGE2) [2, 25], or extracellular matrix components (e.g. versican) [26] shown to be expressed by primary cervical tumor cells, leading to immunosuppressive cell subset accrual and preventing antitumor cell subsets to enter the tumor area.

In order to unveil coordinated suppression through primary tumor-derived lymph flow, we have grouped pelvic lymph nodes according to anatomical location, which showed a unique lymphatic suppressive drainage pattern per patient. Next to the high numbers of Tregs, HLA-DR^+^- and PD-L1^+^ myeloid cells, and low CD8^+^ T cell/Treg ratios in LN+, interestingly, also in a number of LN−, we found high Treg numbers and low CD8^+^ T cell/Treg ratios. The measured PD-L1^+^ cells are most likely M2 (CD163^+^) macrophages [7], originating from monocytes in the presence of IL-6, PGE2 and IL-10 [10, 25].

Also, in lymph nodes located distal from the primary tumor, at the iliaca communis region, the microenvironment might be immunosuppressive as suggested by the here presented data. This might be explained by sentinel lymph node (SLN) identification studies in cervical cancer, in which first-line draining lymph nodes were found proximal, but also in distal locations including the iliaca externa region, the fossa obturator region, the iliaca communis region, and within the parametrium [27, 28]. This indicates that the (immunosuppressive) tumor flow is unpredictable and varies highly per patient. In cervical cancer, only one study found higher CD4^+^/CD8^+^ T cell ratios in proximal lymph nodes compared to distal lymph nodes in relation to the distance from the primary tumor, but for FoxP3^+^ Tregs no significant difference was found [13]. This discrepancy might be explained by the fact that the latter study was performed irrespective of SLN localization and tumor flow.

Interestingly, we found similarly elevated Treg levels and CD8^+^ T cell/Treg ratios in LN+ and adjacent LN−* as compared to LN− at more distant anatomical locations. Our data suggests an environmental switch in these LN−* induced by a draining flow, carrying immunosuppressive factors [6, 29] as well as micro-metastases [30, 31] from the primary tumor and/or metastatic lymph node, possibly pushing the microenvironment towards an immunosuppressive state ahead of metastatic tumor spread. In contrast to Tregs, PD-L1^+^ myeloid cells were more strictly associated with the presence of metastases. We hypothesize that primary tumors convert CD14^+^ monocytes into suppressive PD-L1^+^ M2-like macrophages [10], which are able to induce Treg expansion. Prior to metastasis, lymph nodes are conditioned by these Tregs to form a metastatic niche. Subsequently, metastatic tumor cells again recruit and convert PD-L1^+^ M2-like cells and facilitate the expansion of a next wave of Tregs preparing the way for further metastatic spread.

These observations support previous findings on an improved survival of patients with LN+ after complete lymphadenectomy compared to patients with LN+ with an uncompleted lymphadenectomy [32], and might be important for surgical intervention and the exploration of therapies aimed at counteracting the immunosuppressive microenvironment in the primary cervical tumor and the tumor-draining lymph nodes by checkpoint inhibitors, e.g. anti-PD-L1 to inhibit M2-macrophages or anti-CTLA4 to deplete Tregs, thus inducing a robust antitumor T cell response and breaking the vicious cycle of immune suppression and metastatic spread.

## MATERIALS AND METHODS

### Patient population

In order to study the immune cell subsets in pelvic lymph nodes relative to the distance from the primary tumor, all formalin-fixed paraffin-embedded lymph nodes were collected from different proximal and distal anatomical locations, including fossa obturator, iliaca externa, iliaca communis and parametrium, from five patients with cervical squamous cell carcinoma (SCC) presenting with FIGO stage IB (according to International Federation of Gynecology and Obstetrics), who underwent radical hysterectomy and pelvic lymphadenectomy according to Wertheim-Okabayashi [20] or lymphadenectomy only as primary treatment between 2005–2008 at the Academic Medical Center (AMC; Amsterdam, The Netherlands). Patients were selected on FIGO stage, the presence of lymph node metastases and practicable, total number of dissected lymph nodes (Table 1). Patient samples were handled according to the medical ethical guidelines described in the Code of Conduct for Proper Secondary Use of Human Tissue of the Dutch Federation of Biomedical Scientific Societies.

**Table 1:** Clinical characteristics of the study group

	Patient 1	Patient 2	Patient 3	Patient 4	Patient 5
**Age (in years)**	45	37	45	32	49
**Tumor size (in mm)**	30	60	70	50	70
**Parametrium invasion**	No	No	Yes	No	No
**Vagina involvement**	No	No	No	No	No
**Lymph node numbers**					
**-LN−**	24	16	12	15	10
**-LN+**	2	1	3	2	1
**Type of treatment**	LND + chemoradiation	RH + chemoradiation	RH + chemotherapy	LND + chemoradiation	RH + radiation
**Recurrence 5 year**	Yes	No	No	No	No
**Survival 5 year**	Yes	Yes	Yes	Yes	Yes

LND = lymph node debulking

RH = radical hysterectomy and lymphadenectomy

Additionally, fresh lymph node samples were collected from one cervical SCC patient with FIGO stage IIB, primarily treated with lymph node debulking in the AMC (Amsterdam, The Netherlands). These cells were used for FACS analysis as described below. This study design was approved by the Medical Ethical Committees of the AMC (Amsterdam, The Netherlands). The patient gave written informed consent.

### Immunohistochemistry

In total, 9 LN+ and 74 LN− (from 3 LN− insufficient material was present), were obtained from the archive of the Pathology department at the AMC (Amsterdam, The Netherlands); sectioned at 4 μm and mounted on Starfrost slides (Waldemar Knittel, Germany).

For multicolor immunofluorescence staining of CD8, FoxP3 and HLA-DR, slides were deparaffinized for 3 × 5 min in Neo-Clear (VWR, Catalog# 1.09843.5000), and in series of decreasing concentrations of alcohol (1 × 5 min in 100%, 1 × 5 min in 96%, and in 1 × 5 min in 70%). Subsequently, the slides were placed in Milli-Q water for 5 min, and then antigen retrieval was achieved by 10 min boiling in Tris-EDTA buffer at pH 9.0. Afterwards, the slides were allowed to cool down for at least 45 min, and were washed for 3 × 5 min in PBS. Slides were incubated with a mixture of primary antibodies diluted in 1% BSA/PBS overnight at room temperature (RT); 1:100 mouse IgG1 anti-FoxP3 (Abcam, Catalog# ab20034), 1:75 mouse IgG2b anti-CD8 (Novocastra, Catalog# NCL-CD8-4b11), and 1:500 rabbit anti-HLA-DR (Abcam, Catalog# ab137832). Next, slides were washed for 3 × 5 min in PBS, and then incubated with a mixture of secondary antibodies diluted in 1% BSA/PBS for 1 hour at RT; 1:200 Alexa Fluor 488 goat anti-mouse IgG1 (Life Technologies, Catalog# A21121), 1:200 Alexa Fluor 647 goat anti-mouse IgG2b (Life Technologies, Catalog# A21242), and 1:200 Alexa Fluor 546 goat anti-rabbit (Life Technologies, Catalog# A11010). Next, the slides were washed for 3 × 5 min in PBS, and counterstained with 1:1000 4′,6-diamidino-2-phenylindole dihydrochloride (DAPI), washed in PBS and mounted under coverslips with Mowiol.

For immunohistochemical staining of all 83 lymph node samples for PD-L1, an automated immunostainer (Ventana Medical Systems, Inc. Tucson, USA) was used for deparaffination, antigen retrieval, incubation of the primary antibody 1:200 rabbit anti-PD-L1 (Cell signaling, Catalog# 13684), detection, and visualization steps, according to the manufacturer's instructions. Sections were counterstained with Hematoxylin, dehydrated, and mounted under coverslips.

### Imaging, scoring & analysis

The stained slides were analyzed using a fully motorized digital imaging fluorescence microscope (Axiovert-200M, Zeiss, Germany) or a bright-field microscope (Olympus BX50, Olympus, USA).

From each LN−, three to five representative T cell areas (paracortical areas without B cell follicles) were selected and imaged; from each LN+, three different areas were selected and imaged three to five times; tumor area, peri-tumoral area and paracortical T cell area. All pictures were taken at a 200x magnification. Tumor fields were morphologically distinguished from normal tissue by the use of nuclear staining with DAPI. The area (in mm^2^) of the region of interest was determined using image J (National Institutes of Health (NIH), USA) and SlideBook 5.5 Reader (Intelligent Imaging Innovations (3I), USA). Cell counting of positively stained FoxP3 and CD8 cells from digital images was performed manually using the cell counter function of Image J. Results were expressed as number of cells per mm^2^. HLA-DR^+^- and PD-L1^+^ cells were semi-quantitatively scored because their density and irregular morphology made it difficult to reliably count these cells and therefore we distinguished three groups: (−/+) minimal-, (+) moderate-, and (++) high numbers of positive cells in both LN− and LN+ (see Supplementary Figure 2). For some of the analyses, lymph node groups were categorized as follows: (1) LN+, (2) LN−* (LN− located in the same anatomical localization [i.e. lymph node station] as LN+) and (3) all LN− in the remaining anatomical locations, distal from LN+. In the latter analysis, parametrial lymph nodes were excluded.

### Fresh lymph node collection, processing and flowcytometric analysis

Two lymph node samples were collected from one metastatic lymph node from a cervical cancer patient (see above). An experienced pathologist identified macroscopically two distinct regions: metastatic tumor and pre-existent lymphoid area. From both areas, cells were collected and processed to single-cell suspensions as previously described [7]. To study T cell frequencies, four-color flow cytometry was performed on the single-cell suspensions using a set of antibodies: CD4-FITC, CD3-APC (BD), CD25-APC (BD), CD3-PerCp-Cy5.5 (BD), CD8-PE (all from BD Biosciences, USA), and FoxP3-PE (eBioscience, USA). To identify Tregs (CD3^+^CD4^+^CD25^+^FoxP3^+^), a membrane and intracellular staining was combined as previously described [7]. Mouse-IgG1 and rat-IgG2a antibodies were used as isotype control. Analyses were performed on a BD FACSCalibur (BD) and data were analyzed using CellQuest Pro software (BD).

### Statistical analysis

All statistical analyses were performed using GraphPad Prism 5. The one-way ANOVA Friedman test was used to compare Tregs, CD8^+^ T cells, and CD8^+^ T cell/Treg ratios between three groups (T cell area/peri-tumoral area/tumor area and LN+/LN−*/LN−). Comparisons between specific groups were performed with Dunn's post hoc test. The two-sided Chi^2^ Fisher's Exact test was used to analyze differences between distribution of HLA-DR^+^ and PD-L1^+^ cells, between two groups, and between three groups (T cell area/peri-tumoral area/tumor area and LN+/LN−*/LN−). Kruskal-Wallis test was used for comparison between three independent groups. Results were significant when *P* < 0.05.

## SUPPLEMENTARY FIGURES


