# Supplementary material for: Nodal metastasis in cervical cancer occurs in clearly delineated fields of immune suppression in the pelvic lymph catchment area
Source: Oncotarget. 2015 Sep 28;6(32):32484–93. doi: 10.18632/oncotarget.5398 (PMC4741707; doi:10.18632/oncotarget.5398)
Supplement: Supplementary file 1 [file oncotarget-06-32484-s001.pdf]

SUPPLEMENTARY FIGURES

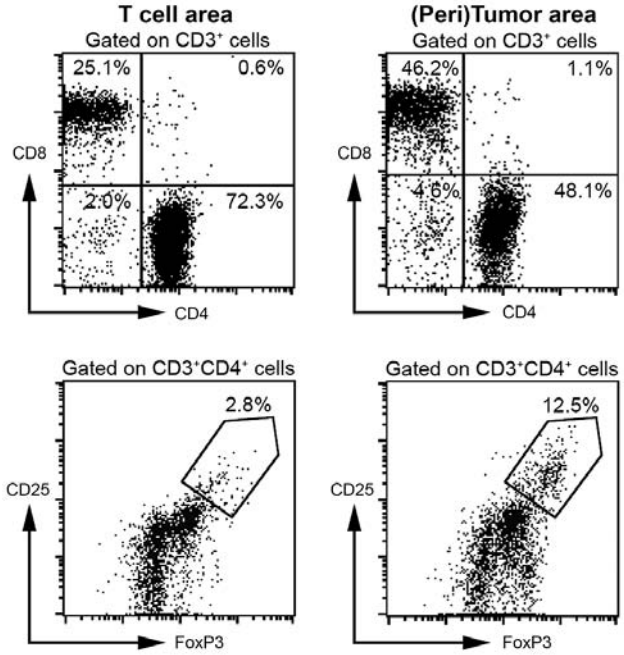

Supplementary Figure S1: Flowcytometric analysis of CD8$^+$ T cell and Treg frequencies in lymphoid vs. tumor tissue from a cervical tumor draining lymph node. A lower percentage of CD8$^+$ T cells (46.2% vs. 25.1%, upper panel), a higher percentage of CD4$^+$ T cells (48.1% vs. 72.3%, upper panel), and less Tregs (identified by CD3$^+$CD4$^+$CD25^{high}FoxP3$^+$ cells) (2.8% vs. 12.5%, lower panel) were found in the lymphoid area compared to the tumor area in one freshly collected lymph node as analyzed by flow cytometry.

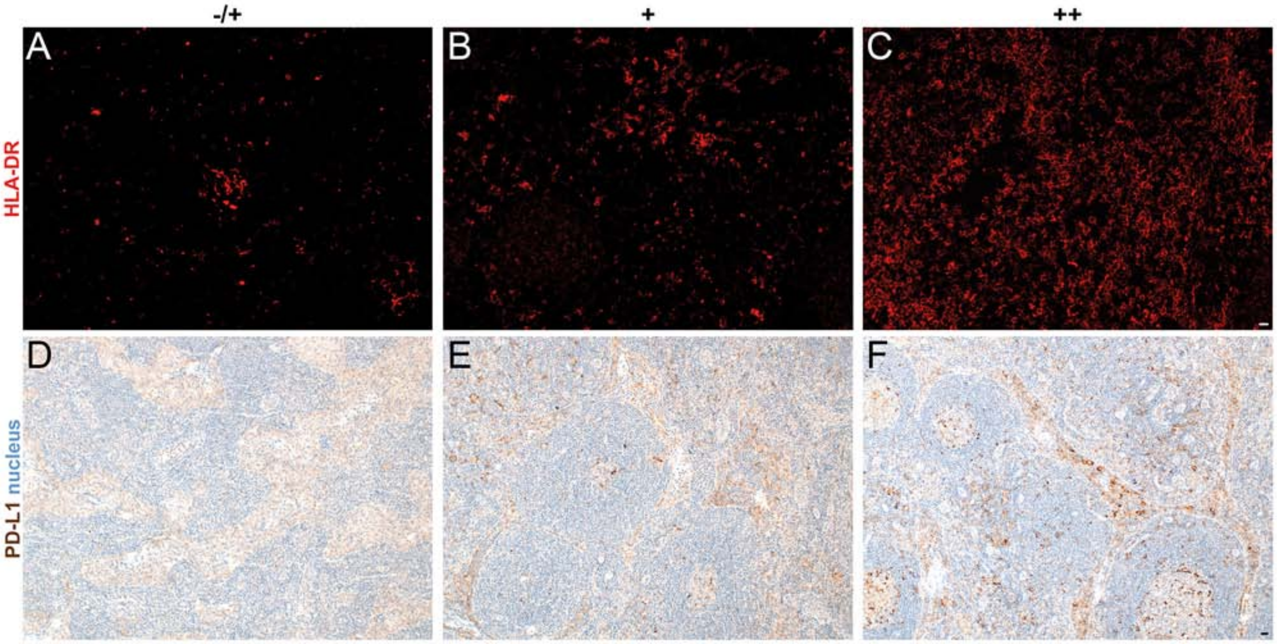

Supplementary Figure S2: Representative images for semi-quantitative analysis of HLA-DR and PD-L1. A. Minimal- (−/+), **B.** moderate- (+), and **C.** high numbers of HLA-DR^{+} cells in lymph nodes from patients with cervical cancer. **D.** Minimal- (−/+), **E.** moderate- (+), and **F.** high numbers of PD-L1^{+} cells in lymph nodes from patients with cervical cancer. NB: in (D) a very weak brown staining is visible in lymph node sinuses which was regarded as background. Scale bars are 20 µm.
